# Lasers, Light, and Energy-Based Devices in Plastic Surgery: A 3-Year Review of a Resident Cosmetic Clinic Experience

**DOI:** 10.1093/asjof/ojae094

**Published:** 2024-11-29

**Authors:** Vidhya Nadarajan, Bhavana Thota, Anca Dogaroiu, Lauren Kim, Amor Niksic, Jennifer Barillas, John Hoopman, Bardia Amirlak, Jeffrey M Kenkel

## Abstract

**Background:**

The increasing utilization of laser and light-based technologies in plastic surgery has heightened the need for comprehensive training programs within residency programs, allowing trainees to remain competitive in the cosmetic medicine field.

**Objectives:**

This review intends to describe our trainees’ experience with lasers in their last 2 years of training while participating in their resident cosmetic clinic.

**Methods:**

This retrospective chart review examines laser procedures conducted from 2021 to 2023 within the Resident Cosmetic Clinic at the study institution. Data were gathered from internal records and patient charts, focusing on demographics, procedure types, complication rates, and training structures.

**Results:**

This study analyzed 162 resident cosmetic laser, light-based device, and radiofrequency cases between 2021 and 2023. There were 90 patients with an average age of 46.8 years with the majority being females (95.6%). Intense pulsed light was the most frequently used modality used in 46.01% of procedures. The overall complication rate was 6.17%.

**Conclusions:**

The findings suggest that resident laser clinics, under proper supervision, provide a safe and effective training ground for future plastic surgeons. This study underscores the need for standardized and comprehensive education to enhance resident knowledge and skills in laser and other energy-based devices.

**Level of Evidence: 3 (Therapeutic):**

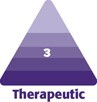

Lasers were introduced to medicine in the 1980s primarily for treating vascular malformations.^1^ Because of their ability to deliver controlled energy to specific chromophores within body tissue, lasers and other energy-based devices offer enhanced outcomes and greater control compared with other treatments.^2,3^ Thus, although initially developed in dermatology, laser procedures are now prevalent in multiple medical specialties, including plastic surgery.^4^ According to The Aesthetic Society's 2022 Aesthetic Plastic Surgery National Databank Statistics, 1,047,794 combination laser skin treatment procedures and a total of 2,758,690 energy-based treatments (hair removal, treatment of vascular malformations, etc) were performed in 2022. The revenue generated from these procedures amounted to $776,058,800 and $1,540,845,902, respectively.^5^ The rise in laser utilization, especially in the field of plastic surgery, underscores the need for comprehensive education for trainees to uphold treatment effectiveness with patient safety and remain competitive.

In 2011, a survey conducted by Oni et al indicated that plastic surgery residency programs offer training in laser resurfacing (83.7%) and noninvasive laser techniques (81.4%).^6^ In 2015, the Accreditation Council for Graduate Medical Education (ACGME) minimum laser case requirements increased from 5 cases (2011-2014) to 10 cases to reflect this change in the realm of aesthetic practices. That year, the bottom 10th percentile of plastic surgery residents did not meet the minimum case requirements for laser procedures, suggesting some residents may be graduating without adequate experience in this area.^7^ Furthermore, in a study, McNichols et al showed only 43% of residents who participated in a cosmetic clinic performed laser procedures.^8^ Thus, it is apparent that the overall experience and training with laser procedures are not standardized or uniform across different programs. Despite the presence of devoted cosmetic clinics, the number of cases performed varies significantly among residents, impacting their comfort and competence with laser procedures.

A comprehensive outline for laser training education for practicing plastic surgeons and trainees has been published by Wamsley et al.^9^ This outline included descriptions of the characteristics of light, an introduction to fundamental laser principles, a comparison of lasers and pulsed light systems, and clinical scenarios in which light-based devices may be utilized.^9^ The information presented in this outline contained the fundamentals of the dedicated didactics that residents at the study institution receive as part of their laser training.^9^ In addition to annual didactic sessions, plastic surgery residents at the study institution receive hands-on training during their last 2 years, actively treating their own patients with faculty oversight in the resident cosmetic clinic. The objective of this study is to review the resident cosmetic clinic experience with regard to their use of lasers and other energy-based devices.

## METHODS

### Study Design and Research Plan

This retrospective chart review was approved by IRB at the study institution. Data collection utilized internal/departmental records/Epic charting over the last 3 years (2021-2023). Cases were identified using billing CPT codes specific to lasers and light-based devices (17106, 17107, 17108, 15780, 15781, 17999, 17260-17280, 15782, 15783. 15788, and 15789). Chart review was conducted to evaluate patients, diagnoses, cases, complications, and outcomes. All patients seen at the Resident Cosmetic Clinic for laser or light-based and energy-based treatments were treated by senior residents (PGY5 and PGY6) and supervised by an experienced faculty and/or laser specialist.

### Clinic Structure

In the laser training program at the study institution, residents have the opportunity to see and treat patients with lasers and other energy-based devices during their last 2 years of training in their cosmetic clinic. Patients seen are discussed with a senior faculty and/or laser specialist, and a plan is developed for treatment. All full ablative procedures are staffed by an experienced faculty member.

### Statistical Methods

Descriptive statistics, including but not limited to mean, median, mode, and standard deviation, were performed for all data points, as appropriate.

## RESULTS

### Patient Demographics

**Figure 1.**
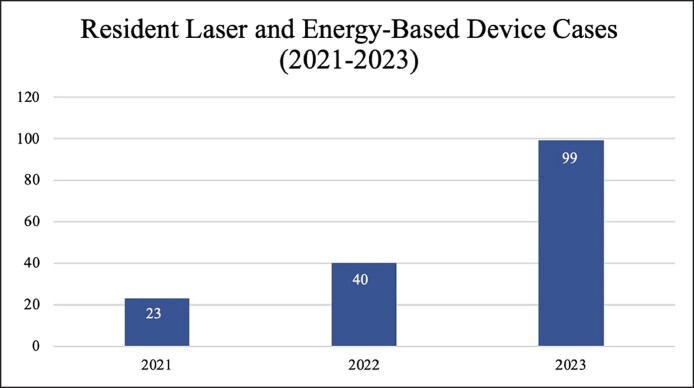
Resident laser and energy-based device cases, 2021 to 2023.

From January 2021 to December 2023, there were 162 laser/light-based device treatments performed at the Resident Cosmetic Clinic (Figure 1). Cases involved 17 residents, 7 faculty members, and 4 aesthetic fellows. There were 90 individual patients who received laser treatments from a resident with an average of 2.07 ± 1.39 visits per patient. The average patient age was 46.8 ± 13.6 years, and the majority were females (86, 95.6%; Table 1).

**Table 1.** Patient Demographics

Demographic Data	*n*
Sex	Female: 86, 95.6% Male: 4, 4.4%
Age, mean ± SD	46.8 ± 13.6
Race	Caucasian: 71, 78.9% Unknown: 14, 15.6% Asian: 4, 4.4% African American/Black/African Descent: 1, 1.1%
Ethnicity	Non-Hispanic/Latino: 68, 75.6% Unknown: 13, 14.4% Hispanic/Latino: 9, 10.0%
Fitzpatrick skin type	I-II: 35, 38.9% Not documented: 34, 37.8% III-IV: 18, 20.0% V-VI: 3, 3.3%

SD, standard deviation.

### Types of Resident Cases

**Figure 2.**
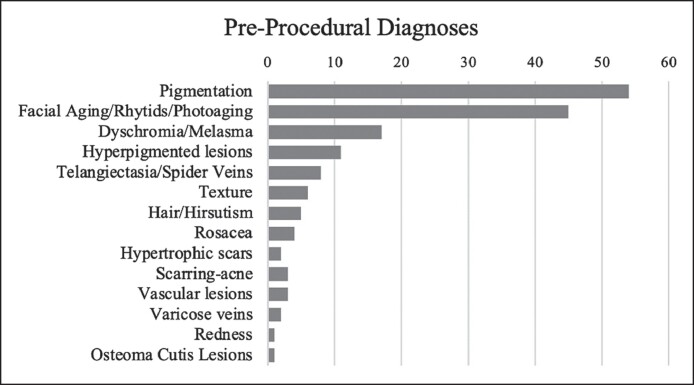
Preprocedural diagnoses for laser and energy-based device treatment.

Pigmentation was the most common presenting concern for individuals seeking energy-based device treatment at the Resident Cosmetic Clinic (Figure 2).

**Figure 3.**
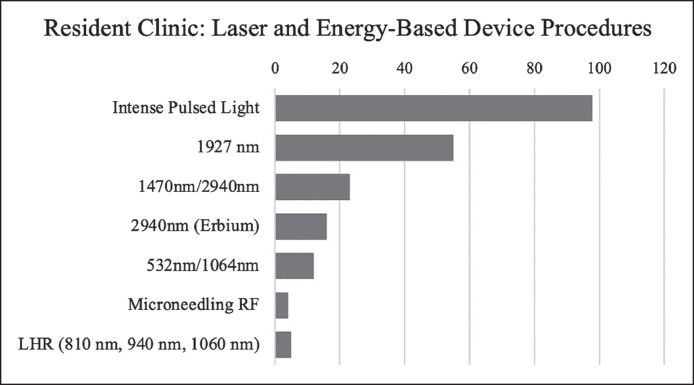
Resident laser and energy-based device procedures. LHR, laser hair removal, RF, radiofrequency.

Intense pulsed light (IPL) was the most frequent treatment (*n* = 98, 46.01%), followed by the fractional 1927 nm (*n* = 55, 25.82%), and the hybrid 1470 nm, 2940 nm laser (*n* = 23, 10.80%). (Figure 3, Table 2). The majority (*n* = 146) of treatments were to the face (Table 3).

**Table 2.** Laser and Energy-Based Device Procedures Among Plastic Surgery Residents

Laser and energy-based device procedures	Resident procedures	
	*n*	%
Intense pulsed light	98	46.01
Nonablative, fractional—1927 nm	55	25.82
Nonablative + ablative—1470 and 2940 nm	23	10.80
Erbium—2940 nm	16	7.51
532 and 1064 nm	12	5.63
Laser hair reduction—810, 940, and 1060 nm	5	2.35
Microneedling—radiofrequency	4	1.88

**Table 3.** Treatment Sites for Laser and Energy-Based Device Cases

Anatomic location	*n*
Face	146
Chest/sternum	26
Neck	17
Submentum	6
Hands	6
Thighs	6
Legs	6
Earlobes	5
Bilateral mandibular	5
Preauricular	3
Back	3
Arms	2

All graduating residents from 2021 to 2023 achieved case minimum requirements for lasers (10 minimum cases). The average number of cases for integrated and independent graduates in plastic surgery per year is available in Table 4. Of note, residents also perform laser treatments for vascular malformations on their pediatric rotation, which are submitted to ACGME under their own section.

**Table 4.** Average Laser and Energy-Based Device Cases for Graduating Plastic Surgery Residents 2021 to 2023 Compared With ACGME Case Minimum Requirement for Lasers

Graduating year	Program	Cases	Case minimum requirement (lasers)
2021	INT	15.7	10
2021	IND	11.5	10
2022	INT	19.3	10
2022	IND	10	10
2023	INT	20.3	10
2023	IND	17	10

ACGME, Accreditation Council for Graduate Medical Education.

### Adverse Events

Among the 162 evaluated cases, 146 (93.8%) had no reported complications. The overall complication rate was 6.17% (*n* = 10), with no major complications reported. All complications were within the known adverse events for the procedures (Table 5).

**Table 5.** Adverse Events Documented Among Resident Laser and Energy-Based Cosmetic Cases

Complication/adverse event	*n*	%
Persistent redness/reddish pigmentation	2	1.2
Rash	1	0.6
Skin sloughing	1	0.6
Swelling	1	0.6
Hyperpigmentation	1	0.6
Acne	1	0.6
Multiple postoperative concerns	3	1.9

The most common minor issue apart from the complications listed above was unsatisfactory improvement managed with a planned repeat procedure which occurred in 12 cases.

## DISCUSSION

With the advancement of laser and light-based technology, plastic surgeons have expanded treatment options for office-based nonsurgical rejuvenation. Lasers have become a useful adjunct to help better serve the needs of patients; thus, it is important for plastic surgery trainees to gain knowledge and experience in lasers, light-based devices, and radiofrequency. In this article, we provide an overview of the resident clinic laser case volumes in order to quantify the average resident's exposure to an increasingly popular modality of treatment.

In a 2008 survey of plastic surgery residents, 54% of residents confirmed receiving training in laser resurfacing, and 35.7% of residents reported receiving training in noninvasive laser treatments.^10^ Additionally, 50% of residents expressed a desire for more training in laser resurfacing. These results suggested an inadequacy of laser training, resident confidence, and satisfaction with training. Efforts to improve this were implemented by programs and by 2011, 66% of residents and 49% of residents reported exposure to lasers resurfacing and noninvasive laser treatments, respectively.^6^ Because lasers continued to gain popularity, the ACGME requirements for lasers were increased to 10 cases minimum for graduation of plastic surgery residency. This number, while doubling the requirement, is still minuscule. Furthermore, despite this change, the level of training among plastic surgery trainees in lasers continued to vary with 1 study, demonstrating a 2.7- to 22.0-fold difference in experience.^7^

The majority of existing literature on laser training for aesthetics in residency are represented in Table 6. In our review, most studies on laser training in residency are survey based, showing increased resident exposure to lasers with interest in laser education among plastic surgery programs.^6-8,10,15^ Residents gain exposure to noninvasive laser treatments and laser resurfacing through cosmetic surgery rotations, resident aesthetic clinics, or both. Additionally, some programs may have dedicated resident laser training, such as didactics to supplement their learning (Table 6). Despite the breadth of literature describing resident laser training in plastic surgery residency, there are no studies detailing the procedures performed and outcomes. Specific procedural details of laser use in residency are limited largely to dermatology publications.^14,16^

**Table 6.** Studies on Resident Training in Lasers

Authors	Year	Structure	Study design	Outcomes results
Morrison et al^10^	2008	Plastic surgery	Survey	Discrepancy between program perception and resident reports on laser training (PDs believe over 75% of residents receive adequate laser training whereas about 50% of residents felt they needed more experience with laser resurfacing)
Oni et al^6^	2011	Plastic surgery	Survey	Resident exposure increased to 66% Increased resident confidence Desire for more training in lasers
Momeni et al^11^	2014	Plastic surgery	Survey	43.5% to 59.0% of senior residents would like to spend more time developing their skills in lasers
Silvestre et al^7^	2016	Plastic surgery	Survey	Major variation in resident laser exposure with 2.7- to 22.0-fold differences in experience
Weissler et al^12^	2016	Plastic surgery	Survey, program-specific training	Residents had 73.9% confidence in performing lasers following graduation
McNichols et al^8^	2017	Plastic surgery	Survey	Only 43% of residents who participated in RCC performed lasers
Wamsley et al^9^	2021	Plastic surgery, LC RCC	Program-specific training	Laser clinic Resident cosmetic clinic Didactic sessions, including residents of all levels
Marks et al^13^	2022	Plastic surgery, LC RCC	Program-specific training	Training Nonsurgical/laser clinic (quarterly) Residents of all levels attend, yearly
Desai et al^14^	2023	Dermatology RCC	Program-specific training	Training, case volume Monthly didactic sessions and hands-on training Pre- and postprocedure photography for teaching and reviewing patient progress

This is the first study, to date, to report the outcomes and details of laser, light-based, and radiofrequency procedures in a resident cosmetic clinic in plastic surgery. Our findings support previous claims that including lasers in resident clinics helps residents meet case minimums for aesthetic procedures and educates them on a treatment option many will consider while in practice.^15^ For the past 3 years, all graduating chief residents have exceeded the minimum case requirements for laser procedures, with the average graduating integrated plastic surgery resident in 2023 completing double the minimum requirement (Table 4).

At our institution, chief and senior residents see their own patients with faculty oversight, as part of the resident cosmetic clinic in which they offer aesthetic procedures, such as surgery, injectables, and lasers at a discounted rate with faculty/expert supervision.^13,17^ The most frequently used devices in this clinic were IPL, the fractionated 1927 nm, and the hybrid 1470/2940 nm laser. IPL is commonly used in skin rejuvenation or addressing vascular lesions, pigmentation, and hair removal. The nonablative, fractional 1927 nm has shown efficacy in treatment for melasma in Fitzpatrick skin types I to IV.^18^ The hybrid laser combines nonablative and ablative wavelengths at 1470 and 2940 nm for skin resurfacing, fine lines, and wrinkles. Residents also gained experience with lasers operating at 532 and 1064 nm wavelengths to treat vascular lesions, pigmented lesions, and pigment for tattoo removal. Other procedures performed by our trainees include energy-based microneedling and laser hair reduction using wavelengths at 810, 940, and 1060 nm to target melanocytes.^9^

This breakdown of the most frequently used devices in our resident cosmetic clinic mirrors the most commonly used laser devices and treatments performed by practicing plastic surgeons, further supporting the role of laser and light-based device cases in allowing residents to remain competitive and knowledgeable in cosmetic medicine. Per The Aesthetic Society's 2019 Aesthetic Plastic Surgery National Databank Statistics, 99,740 facial photorejuvenation treatments using IPL and 180,332 hair removal treatments using laser or pulsed light were performed in 2019.^19^ Laser treatments have continued to remain popular with The Aesthetic Society's 2022 Aesthetic Plastic Surgery National Databank Statistics listing laser skin resurfacing performed with combination lasers as the most common energy-based device procedure done in 2022.^5^

Certainly, this increased popularity in laser and light-based device treatments is also reflected in the growing volume of resident cases year by year (Figure 1). Over the 3-year period included within this study, the number of laser and energy-based device cases performed by residents increased by 330%. This may be a reflection of the rise in popularity of these treatments among the general population but may also have to do with the study institution's robust practice and dedicated Clinical Center for Cosmetic Laser Treatment, wherein a large number of laser-based treatments are performed by both attending physicians and aestheticians. These patients, or others referred by these patients, may ultimately choose to undergo a few treatments at the resident cosmetic clinic for the reduced fee. Furthermore, the study institution's laser training program has an early emphasis on the multifaceted treatment capabilities of laser and energy-based devices and allows residents to work with an experienced medical laser specialist well-versed in laser physics, safety, and tissue interaction. This likely plays a role in the residents’ comfort in offering laser and energy-based device treatments to address their patients’ aesthetic concerns.

In addition to hands-on training through the resident cosmetic clinic, residents gain exposure to laser treatments for various indications such as pediatric vascular malformations, facial aging, and pigmentation while on the pediatric rotation. This clinical experience is supplemented with two 4 h dedicated didactic sessions annually with hands-on training for residents of all levels.^9^ Although this model serves as an illustrative example of 1 possible education program, we recognize that this is not possible in many institutions for a variety of reasons. For those programs, it may be beneficial to develop an educational relationship with community providers interested in teaching laser, light, and energy-based device procedures.^17^

The essential didactic components of the laser training program include a foundation on the basics of light and laser principles, differentiating between lasers and pulsed light systems, education on treatment parameters (wavelength, power, spot size, pulse width, and cooling), clinical applications, and safety training.^9^ This formal instruction is necessary for operating lasers and light-based devices to ensure the safety of the operator, patient, and care team.

Our clinic had a very low complication rate which we hypothesize can be attributed to the rigorous laser training our residents receive and the oversight provided by our laser specialist and/or faculty. The laser specialist is responsible for guiding residents through laser and other energy-based device procedures and providing insight into the interaction of these devices with tissue. Resident didactics and training sessions are overseen by the laser specialist alongside faculty members. In addition to a strong emphasis on understanding the aforementioned treatment parameters, these training sessions include developing an understanding of proper patient selection, how individual patient factors may impact treatment settings, and posttreatment instructions for minimizing complications. That there were only 10 complications, all known adverse events, among the 162 cases included in this study emphasizes the importance of a thorough understanding of treatment parameters and how to tailor them to individual patients. For example, about 4 patients, all treated with IPL, were counseled in the first visit about the need for a planned repeat procedure in order to achieve their desired results; the staged method utilized for these patients may have prevented adverse events by allowing the resident to utilize more appropriately conservative treatment settings.

Ultimately, dedicated and early didactics in laser and energy-based device treatments and resident cosmetic clinics with the ability to perform these treatments under supervision of a laser specialist are keys to ensuring successful resident training in these increasingly popular treatments. The addition of a dedicated laser staff person—in this case, our aforementioned laser specialist—has opened up the opportunity for residents to perform a greater number of laser cases than could be achieved by oversight from intermittent faculty. For program directors interested in increasing their trainees’ exposure to these procedures, we recommend early emphasis on foundational principles of laser physics and laser–tissue interaction and the ability to then put these principles in action with hands-on learning like that provided by the resident cosmetic clinic experience described within this paper. Supplementing these experiences with dedicated faculty interested in teaching laser, light, and energy-based procedures is crucial, either within the institution or at partnering community practices.

Our study has several limitations that warrant further consideration. The retrospective design of the study relies on the accuracy and completeness of existing records. Previous retrospective studies on resident cosmetic clinics have excluded nonsurgical procedures in their analysis of resident cosmetic clinics because of documentation inconsistencies.^12^ To address this, we cross-referenced billing data for procedures. However, we only included select CPT codes that were specific to cosmetic procedures offered by the resident cosmetic clinic. Data of resident-performed procedures may not have been captured if a procedure was billed to an area other than the resident clinic. Additionally, complications may not have been fully captured, as patients might have sought treatment for adverse events from an attending physician's clinic or another institution rather than through the resident clinic. Furthermore, when comparing with the submitted case numbers for lasers for the ACGME requirements, we note that the submitted case numbers are lower than actual case volume. This is because of some laser procedures being submitted under other categories, such as treatments for vascular malformations with congenital head and neck defects. Also, during our annual “hands-on” laser didactic sessions, residents earlier in their training perform procedures that may not have been accurately captured as this review focused on the latter 2 years of training. Additionally, most of our procedures are done with several residents involved so mathematically, there may be existing differences. Other limitations of our analysis include Fitzpatrick skin type, which was documented in ∼60% of cases, limiting our ability to comprehensively assess patient demographics, outcomes, complication rates based on skin type. These findings are based on data from a single institution, which may limit the generalizability of the results to other plastic surgery residency programs with different structures, resources, patient demographics, and geographic location.

## CONCLUSIONS

Resident laser clinics, under proper supervision, provide a safe and effective training environment for future plastic surgeons. Our study demonstrates that with structured training programs, residents can achieve and exceed the minimum case requirements set by the ACGME, enhancing their competency in laser and light-based procedures while ensuring patient safety.

This study underscores the need for standardized laser training in plastic surgery residency programs to prepare residents for the evolving demands of aesthetic and reconstructive practices. Implementing comprehensive educational guidelines, including both didactic sessions and hands-on training, can significantly improve resident proficiency and confidence in performing laser procedures. Data is available by request.
